# Graphene-catalyzed formation of C≡N bonds via cleavage of C-C and N-O bonds in ethanol and nitrate under room temperature

**DOI:** 10.1038/s41598-018-20238-y

**Published:** 2018-01-29

**Authors:** Ding Xiao, Wucong Wang, Yanzhe Gai, Yaping Zhao

**Affiliations:** School of Chemistry and Chemical Engineering, Shanghai Jiao Tong University, Shanghai, 200240 P. R. China

## Abstract

The cleavage of carbon-carbon bonds and the formation of carbon-nitrogen bonds play crucial roles in chemical synthesis. However, these reactions usually proceed at high temperature and involve multiple steps. Herein, we report an unusual and novel reaction catalyzed by graphene. The C-C bond in ethanol and the N-O bond in nitrate can be broken under room temperature, accompanied by the formation of the C≡N bond. We demonstrate these reactions and elucidate their mechanisms by verifying that the product is silver cyanide which was formed when mixing a solution of silver nitrate and ethanol with graphene dispersion in ethanol at room temperature. The pivotal reason for the reaction is the formation of the precipitated silver cyanide. In a broader context, this discovery opens a significant new path for the breakage of the C-C bond in ethanol and the synthesis of nitriles under mild conditions. Also, the graphene was first reported as a catalyst for the room-temperature reaction.

## Introduction

The formation of carbon-nitrogen bonds and the cleavage of carbon-carbon bonds play essential roles in chemical and pharmaceutical industries, and hence they are attracting increasing attention and intensively studied for chemical synthesis^1–6^. Many achievements have been obtained regarding the C-N single bond construction and its reaction mechanism, especially in aromatic amines^7–13^. The most popular catalytic cycle mechanism is believed to include four necessary steps: oxidative addition, reductive elimination, insertion, and metallization. However, the formation of the C≡N bond under mild conditions has not been reported yet. Also, the cleavage of C-C bonds is relatively difficult due to its considerable bond energy^14–17^, which can be seen in the hardness of diamond and the tensile strength of carbon fibers^18,19^. The most efficient and straightforward method for activating the C-C bonds in aromatic hydrocarbons is to use transition metals as catalysts until now^20–23^. However, the activation of C-C bonds in ethanol by organic synthesis, especially at mild conditions, has not been reported. The break of the C-C bond in ethanol, as a sustainable and carbon-neutral transportation fuel, is pivotal to develop direct ethanol fuel cells with high energy density^24–26^. However, its potential application is limited by the almost-exclusive oxidation of ethanol to acetic acid^15^.

During fabricating a composite of graphene and silver, we found that an unexpected compound was formed as soon as mixing silver nitrate ethanol solution with graphene at room temperature. After intentionally designing this process and identifying the resultant product, we found that a series of exciting and relevant reactions occurred. A new path of the cleavage of the C-C bond was achieved at room temperature, accompanied by the formation of the C≡N bond. The graphene triggered the reaction and was converted entirely into one-dimensional scroll itself. Herein, we present this new chemical incredible reaction and the relevant mechanism, which is a significant and breakthrough discovery.

## Results and Discussion

We intentionally designed a series of experiments to explore the fantastic chemical reaction occurred as soon as mixing silver nitrate ethanol solution with graphene at room temperature. An ethanol solution of silver nitrate and an ethanol dispersion of the graphene were used as starting materials, and the resultant compounds were characterized regarding its structure, elemental composition, and morphology. Accordingly, the appropriate reaction mechanism was proposed.

**Figure 1:**
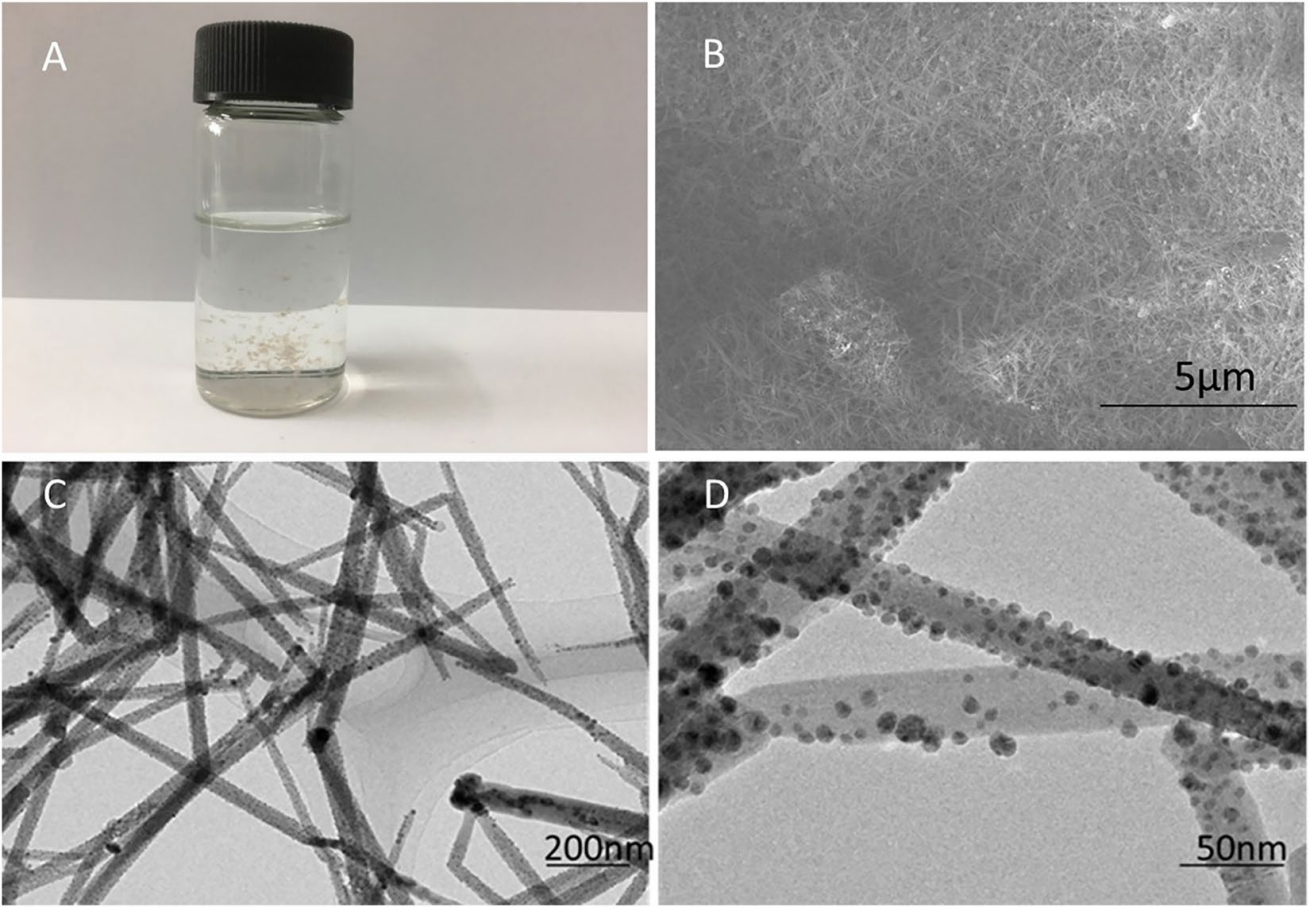
Photos of the resultant product. (**A**) Digital photograph of the product in ethanol solution; (**B**) SEM image; (**C**) and (**D**) TEM images.

The solution of silver nitrate and ethanol was mixed with the graphene dispersion in ethanol by magnetical stirring at 25°C for 15 minutes. The grey precipitates were generated during stirring as shown in Fig. 1A. The resultant precipitates were obtained by filtration, which has a fluffy appearance. The image of the scanning electron microscope (SEM) shown in Fig. 1B displays that the precipitates consist of a pile of thin wires whose length are around three micrometers, and on which there are a significant amount of nanoparticles. The image of the transmission electron microscope (TEM) shown in Fig. 1C further exhibits that these particles uniformly distributed along with the nanowires. The magnification TEM image shown in Fig. 1D indicates the diameter of the wires is around 50 nm, and the particles anchored the nanowires are approximately 10 nm.

**Figure 2:**
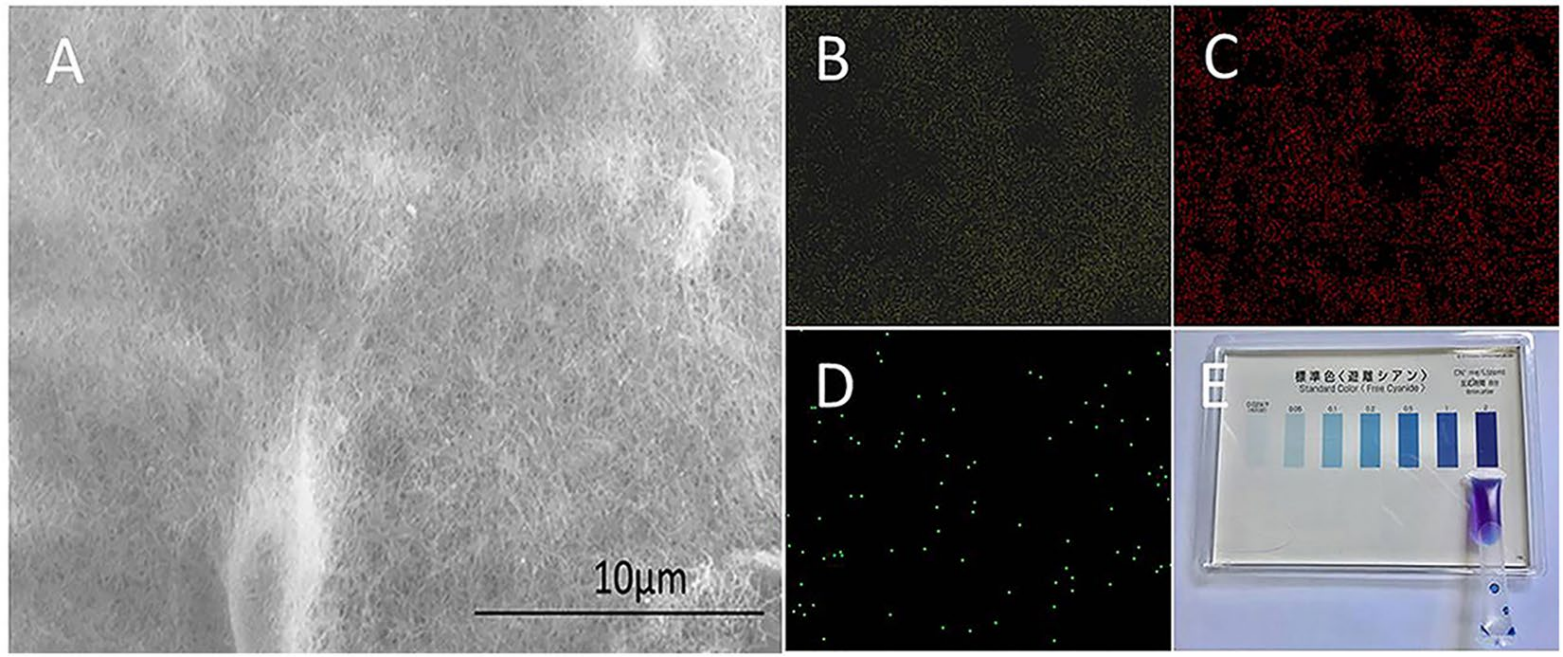
Micromorphology and elemental analysis of the resultant product: (**A**) SEM image; The mapping of energy dispersive spectrometer: Ag (**B**), C (**C**) and N (**D**); Identification of cyanide ion: (**E**) Digital photo of the solution tested by water quality test kit.

To identify the precipitates and elucidate the reaction mechanism, we applied a series of ways to analyze their elemental composition and distribution (see supplementary information). The obtained elemental mappings of Energy Dispersive Spectrometer (EDS) display the distribution of silver, carbon, and nitrogen evenly in the as-produced sample as shown in Figs 2B–D. The selected area of the sample for EDS is shown in the SEM image (Fig. 2A). The nanowires have been identified to be carbon rod which was resulted from the scrolling of the graphene. This dramatic conversion of the graphene will be discussed in a future article. When the nanoparticles were tested using a water quality test kit (see supplementary information), we found that the color of the tested solution appeared dark blue (Fig. 2E) which implies that the nanoparticles contain cyanide ions. To exclude the possibility of the cyanide ions coming from the reactants, we tested the graphene, the silver nitrate, and the ethanol, respectively, using the water quality test kit. All of them show negative reactions as indicated in Fig. S2, which suggests that the reactants did not contain cyanide ions. To confirm that the cyanide ions resulted from the reaction caused by the graphene, we designed three sets of contrast experiments using water, graphite and active carbon, respectively, as the reactants instead of the graphene. We did not find the occurrence of the reaction when the water, the graphite, and the active carbon were used as the reactants, respectively. Also, the test of the water quality test kit for them shows negative results (Fig. S3), which suggests that there are no cyanide ions in the samples. Accordingly, we believe that the graphene plays a crucial catalytic role in the formation of cyanide ions.

**Figure 3:**
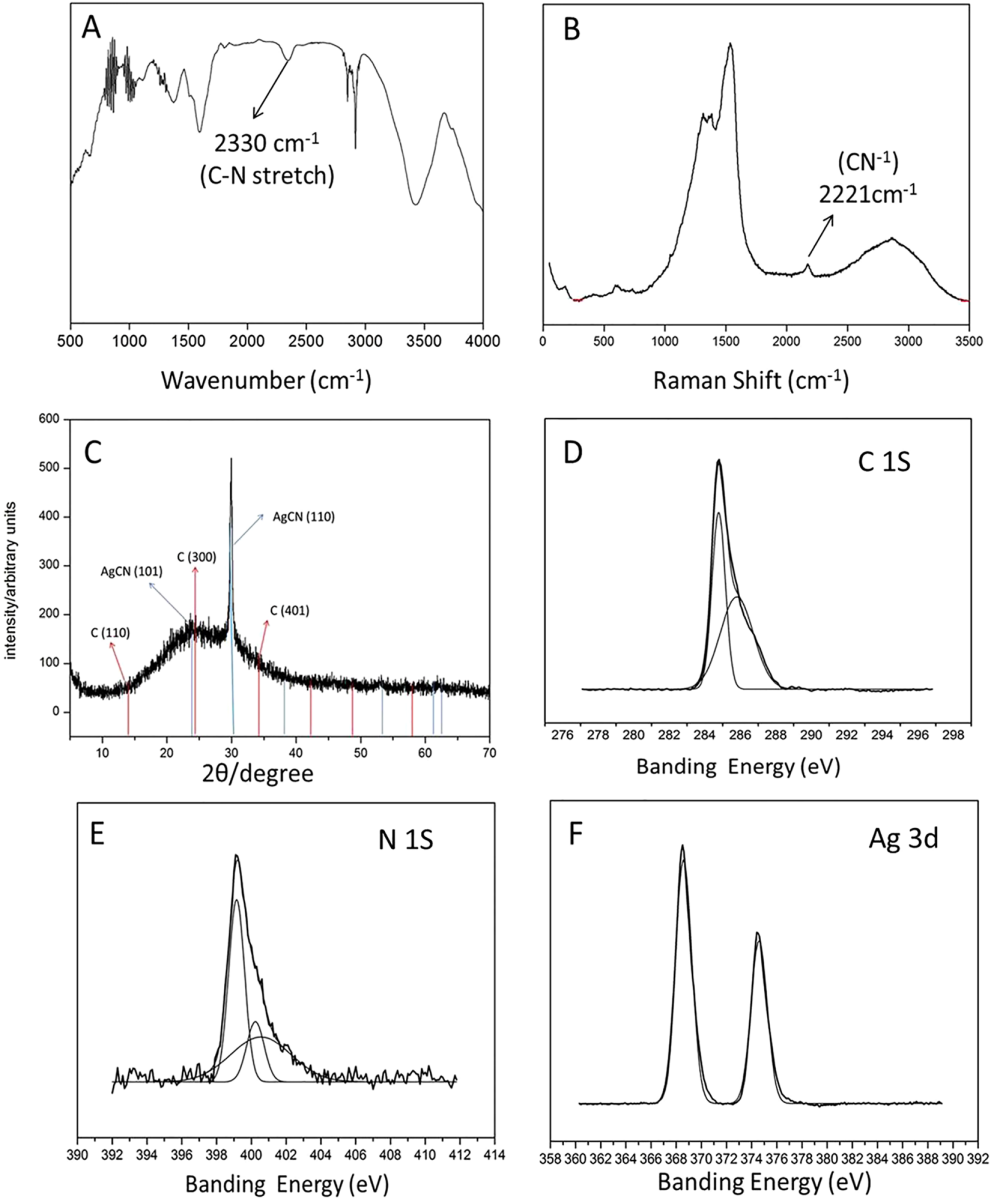
Structural analysis of resultant reaction products. (**A**) FTIR spectra. (**B**) Raman spectra. (**C**) XRD spectra (JCPD standards, C, JCPDS no. 50–0926. AgCN, JCPDS no. 23–1404). (**D**) XPS spectrum of C 1 S photoelectrons. (**E**) XPS spectrum of N 1 S photoelectrons. (**F**) XPS spectrum of Ag 3d photoelectrons.

Combining the results of the water quality test kit and the EDS mapping, we think that the nanoparticles might be silver cyanide. To confirm this speculation, we further analyzed the structure of the nanoparticles by using Fourier Transform Infrared Spectrometer (FTIR), Raman spectra, X-ray diffraction spectra (XRD), and X-ray Photoelectron Spectrometer (XPS). The characteristic absorption peak at 2330 cm^−1^ shown in Fig. 3A, corresponding to the C≡N stretch, suggests that the nanoparticles contain CN^−^. In the same way, Raman spectra (Fig. 3B) indicate that the nanoparticles comprise CN^−^ too because the peak at 2221 cm^−1^ represents the C≡N stretch. The XRD patterns shown in Fig. 3C further confirm that the nanoparticles are silver cyanide. The diffraction peaks at 2θ values of 24.05° and 29.80° suggest the presence of AgCN (101) and AgCN (110) planes, respectively. The diffraction peaks at 2θ values of 15.16°, 22.75°, and 34.29° indicate the presence of C (110), C (300) and C (401) planes, respectively. Also, XPS analysis reveals that the resultant is silver cyanide. The C 1 s peak at 286.3 eV and the N 1 s peak at 399.2 eV shown in Fig. 3D and Fig. 3E, respectively, can be attributed to the CN^−^ ions^27,28^. The C1s peak at 285 eV is assigned to the graphene in the resultant^28^. The Ag 3d peaks at 367.8 eV and 373.8 eV shown in Fig. 3F are assigned to Ag^+^^ 29^. In conclusion, we have proved that the nanoparticles are AgCN.

**Figure 4:**
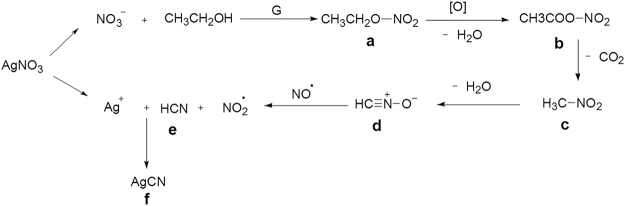
Schematic of the formation mechanism of silver cyanide.

The characterization results aforementioned demonstrate that the silver cyanide was formed when mixing a solution of silver nitrate and ethanol with graphene dispersion in ethanol at room temperature. This reaction involves the cleavage of the C-C bond in ethanol and the break of the N-O bond in nitrate under such mild condition. Given that graphene sheets are not only a kind of carbon nanomaterials but also a type of 2D macromolecules with highly reactive-surface, and they can afford electrons to occupy the vacant orbital of the silver ions, herein, we propose a mechanism to explain the formation of the silver cyanide as shown in Fig. 4. The graphene as a catalyst triggers the reaction of the nitrate with the ethanol forming nitrooxy-ethane (a) like a typical esterification reaction. The activity of the hydrogen in ethanol is enhanced due to the action of a withdrawing electron group. Thus, nitrooxy-ethane is readily oxidized by a strong oxidizing nitrate ion, and a molecule of water is removed to form an acetic nitrous anhydride (b). Also, due to the bonding of a withdrawing electron group, the ester group can easily create nitromethane (c) by removing a carbon dioxide molecule. Nitrile oxides (d) are reactive intermediates which can be generated *in situ* by the dehydration of the nitromethane compound. Both the deoxygenation and dimerization of the nitrile oxides were reported in the previously published reference^30^. Although the deoxygenation reaction from the nitromethane (c) to the nitrile oxides (d) is a reversible process, the formation of the precipitated silver cyanide (f) drives the reaction equilibrium between (c) and (e) toward the direction of (e), which significantly accelerates all the reaction process. The nitrile oxides (d) react with NO was reported in the previously published papers^30,31^. We applied other solvents and nitrates instead of the ethanol and AgNO_3_, respectively, to do the contrast experiments. The results support the mechanism too as shown in Fig. S4–S5. Therefore, the formation of C≡N bonds was achieved via the cleavage of the C-C bond in the ethanol and the N-O bond in the silver nitrate. It is the graphene that triggers the reaction to proceed at room temperature, and it is converted into scrolls itself simultaneously.

## Conclusions

We have demonstrated that the C-C bond in ethanol and the N-O bond in silver nitrate can be broken and formed into the C≡N bond under room temperature. The graphene triggers the reaction, and the formed silver cyanide drives the reaction equilibrium toward the ending direction entirely. The graphene itself was converted into one dimension scrolls. This work opens a new path for the breakage of the C-C bond in ethanol and the synthesis of nitriles under mild conditions and shows that graphene has excellent potential as a catalyst.

## Electronic supplementary material

Supplementary Information
