# Supplementary material for: Graphene-catalyzed formation of C≡N bonds via cleavage of C-C and N-O bonds in ethanol and nitrate under room temperature
Source: Sci Rep. 2018 Jan 29;8:1750. doi: 10.1038/s41598-018-20238-y (PMC5789086; doi:10.1038/s41598-018-20238-y)
Supplement: Supplementary file 1 — Supplementary Information [file 41598_2018_20238_MOESM1_ESM.pdf]

Supplementary Information

Graphene-catalyzed formation of C≡N bonds via cleavage of C-C and N-O bonds in ethanol and nitrate under room temperature

Ding Xiao, Wucong Wang, Yanzhe Gai, Yaping Zhao*

School of Chemistry and Chemical Engineering, Shanghai Jiao Tong University,

Shanghai 200240, P. R. China

*Corresponding author: E-mail:ypzhao@sjtu.edu.cn

Materials and Methods

Graphite powders (SP), Hydrochloric acid (AR), Sodium hydroxide (AR) and Silver nitrate (99.80%) were purchased from Sinopharm Chemical Reagent Co., Ltd., Shanghai, China. Carbon dioxide (99.90%) was obtained from Shanghai High-tech Co., Ltd., China. Ethanol (99.50%) was obtained from Changshu Yangyuan Chemical Co., Ltd., China. Water quality test kit was purchased from Kyoritsu Chemical-Check Lab.

Graphene dispersion in ethanol

Five milligrams of graphene, which was freshly-made at the lab, was dispersed in 300ml of ethanol by ultrasound for 3 min.

A solution of silver nitrate and ethanol

Fifteen milligrams of silver nitrate was dissolved in 100 ml of ethanol.

Identification of cyanide ion using water quality test kit

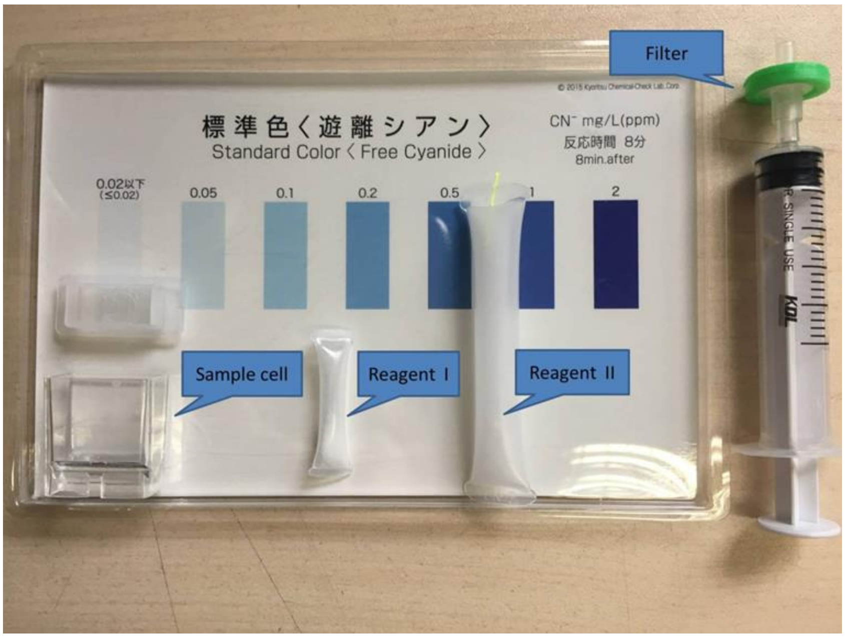

Fig. S1. Digital photographs of the water quality test kit.

One of the methods to detect whether the resultant product contains cyanide ion is to

use the water quality test kit. First, the product is dissolved in dilute hydrochloric acid. Then, the solution is titrated to neutral by sodium hydroxide. After that, the solution is filtered to the sample cell to which reagent I is added. Finally, the solution is transferred to the tube containing reagent II for color reaction to determine the concentration of the solution by comparing its color with the standard color. As shown in Fig.S1, the concentration range varies from below 0.02 PPM to 2 PPM.

Morphology characterization of resultant reaction product

A certain amount of the resultant reaction product was dispersed in a certain amount of ethanol by water bath ultrasound. Then, the dispersion was directly dropped on a silicon substrate, and the carbon film coated copper grids, respectively. After drying under an infrared lamp, the samples were observed by Scanning electrode microscope (SEM) and Transmission electron microscope (TEM), respectively. SEM images were obtained using an FEI Nova NanoSEM 450 (USA). TEM images were captured using JEOL JEM-2100 TEM (Japan) at an acceleration voltage of 120 kV.

Identification of resultant reaction product

The structure of the resultant product was analyzed using various instrumentals for mutual confirmation. Infrared ATR spectra were taken using a Perkin-Elmer Spectrum 100 FTIR instrument, equipped with an ATR sampling accessory. Raman spectra were acquired in Thermo Fisher Scientific with 532 nm laser excitation. X-ray spectroscopy was carried out on Bruker D8 Advance; the spectra were collected between 2θ of 5° and 80°, the scan speed is 6° per minute. EDX was made on Nova NanoSEM 450 instruments, operated with an electron kinetic energy of 12 kV. Surface analysis of the samples was performed with X-ray photoelectron spectroscopy (XPS, *1/AXIS UltraDLD).

Detection of cyanide ion of reaction material

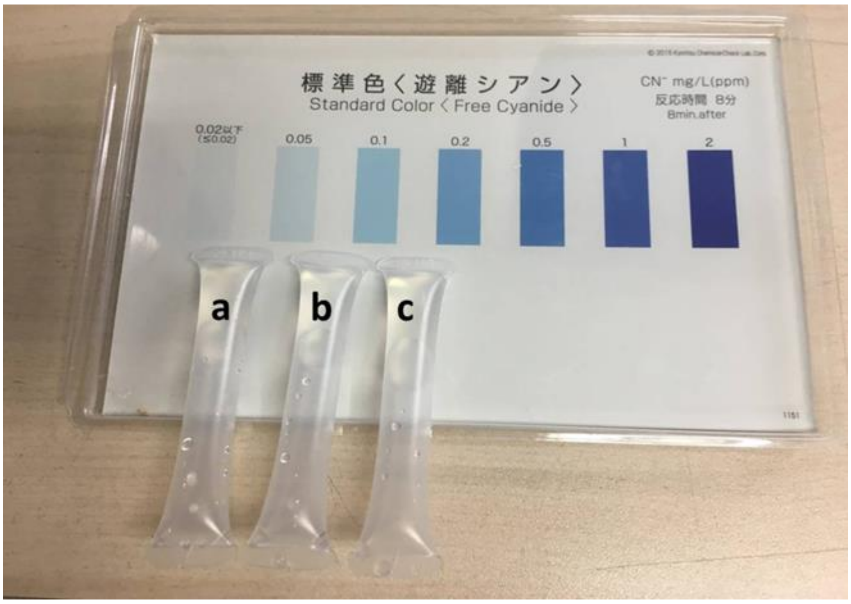

Fig. S2. Digital photograph of samples tested by water quality test kit: (a) graphene, (b) silver nitrate and (c) ethanol.

We detected the starting materials (graphene, silver nitrate, and ethanol) using a water quality test kit regarding cyanide ion. Comparing the color of the solution made from graphene, silver nitrate, and ethanol corresponding to Fig.S2 (a, b, and c), respectively, with that of the standard test solution we believe that the reactants do not contain cyanide ion.

Catalytic effect of graphene

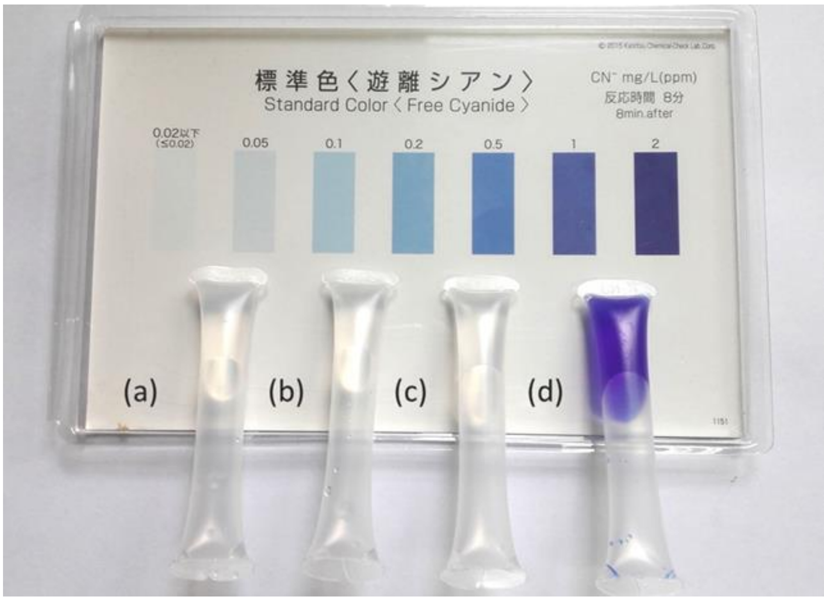

Fig. S3. Digital photo of the tested solution using the water detection kit. (a) water, (b) activated carbon, (c) graphite, (d) graphene.

We designed four sets of experiments to examine the effects of different materials on the formation of cyanide ion. We selected water, activated carbon, graphite as contrast materials. Fig. S3 shows that the solution made from water, activated carbon, and graphite (a, b, and c) except graphene (d), are colorless. It suggests that there is no cyanide ion of the solution made from water, active carbon, and graphite. Therefore, we confirm that activated carbon and graphite cannot trigger the reaction but graphene.

Effects of different reaction solvents on the formation of cyanide ion

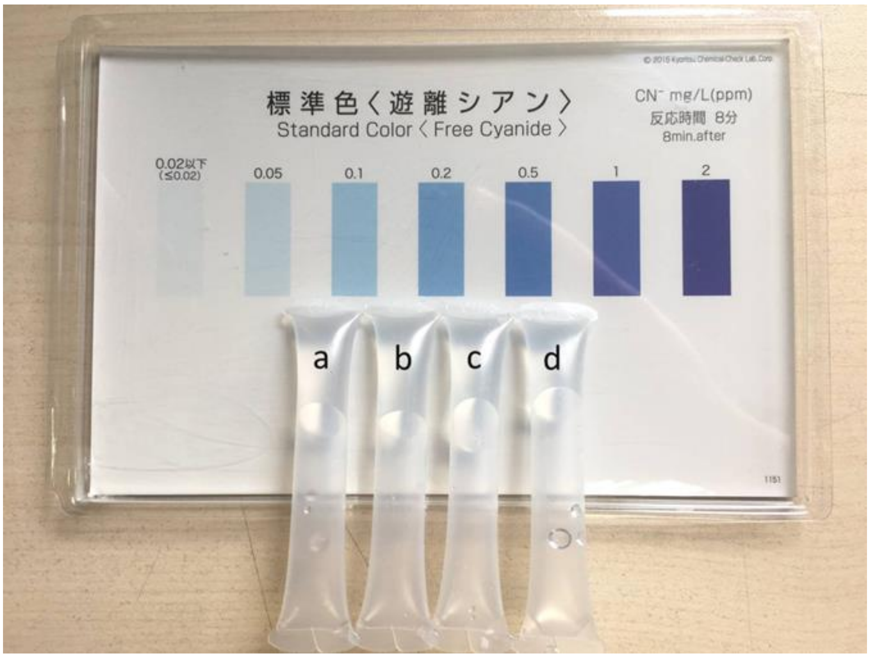

Fig. S4. Digital photographs of the color change of cyanide ion solution in the water detection kit with different solvents, (a) n-hexane, (b) acetone, (c) tetrahydrofuran, (d) propanol.

To verify whether other solvents can react with the silver nitrate in the presence of graphene, we designed a series of experiments with different solvents instead of ethanol using the same tested method as aforementioned. It can be seen from Fig.S4 that there is no change in color for the tested solution prepared by n-hexane, acetone, tetrahydrofuran, and propanol. It suggests that these solvents could not create the reaction.

Effects of different nitrates on the formation of cyanide ion

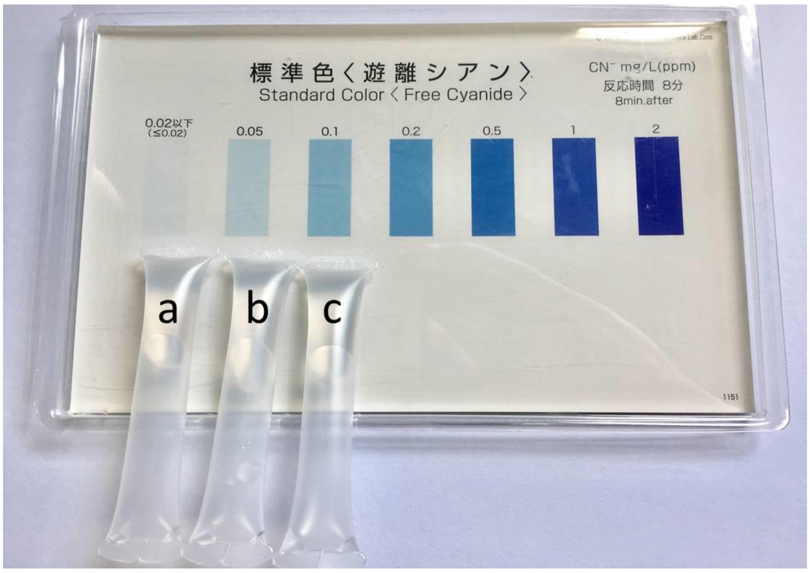

Fig. S5. Digital photographs of the color change of the solution in the water detection kit with different nitrates: (a) Cu (NO_3)$_2$, (b) Ba(NO_3)$_2$, (c)$NaNO_3$.

Other nitrates were applied to study if they could have the same reaction as $AgNO_3$. It can be seen from Fig. S5 that there is no color change for the solution obtained with $Cu(NO_3)_2$, $Ba(NO_3)_2$, and $NaNO_3$. It suggests that these nitrates could not create the reaction.
